# Clostridium difficile infection at a geriatric acute-care hospital in Switzerland between 2008 and 2014: a retrospective cohort study

**DOI:** 10.1186/2047-2994-4-S1-O35

**Published:** 2015-06-16

**Authors:** D Pires, V Sauvan, V Zanichelli, V Prendki, J Reny, S Harbarth, B Huttner

**Affiliations:** 1 Infection Control Unit, Geneva University Hospitals, Geneva, Switzerland; 2 Division of Internal Medicine and Rehabilitation, Geneva University Hospitals, Geneva, Switzerland; 3 Division of Infectious Diseases, Geneva University Hospitals, Geneva, Switzerland

## Introduction

*Clostridium difficile* infection (CDI) epidemiology has changed with an increase in incidence and severity, particularly among the elderly.

## Objectives

We aim to describe CDI epidemiology and assess the effect of infection control measures in the geriatric hospital of Geneva University Hospitals.

## Methods

Retrospective cohort study of CDI patients, identified via active surveillance of the infection control program, at a 300 bed acute-care geriatric hospital in 2008-2014. We analyzed CDI incidence, surveillance case definition (hospital-onset healthcare facility–associated, HO-HCFA), recurrence, demographics, antibiotic exposure, time at risk and in-hospital mortality. Positive tests ≤14 days of diagnosis were excluded. Cell cytotoxicity test was replaced by PCR detection of *C. difficile* toxin B in 01/2010. Infection control measures included contact precautions, environmental cleaning and single room policy. Overall antibiotic use was also analyzed.

## Results

We identified 231 CDI cases resulting in mean incidence of 92.1 cases/10000 admissions. 194 cases (83.9%) were HO-HCFA. Mean age was 85 (+-7.3) years; 26.4% (n=61) were male. Mean CDI incidence (cases/10000 admissions/year) was 124.7(2008), 82.9(2009), 107.9(2010), 100.4(2011), 139.5(2012), 42.9(2013), 46.5(2014). Mean time at risk was 23.5 (IQR 6-34) days. 180 patients (77.9%) received any antibiotics and 59.3% (n=137) ”high-risk” antibiotics in the previous month. 46 patients (29.9%) had a recurrence at a mean of 25 days. In-hospital mortality was 34.4% (n=76). From 01/2011 to 03/2013 single-room policy for CDI cases was suspended. During this period CDI incidence was 115.6 cases/10000 admissions, compared to 39.2 cases/10000 admissions after policy reinstitution (IRR 3.02, 95% CI 2.01-4.53). Overall antibiotic use slightly decreased from 260 DDD/1000PD in 2008 to 218 DDD/1000PD in 2013.

## Conclusion

We observed a low incidence of CDI at the acute care geriatric hospital after a peak in 2012. The decreasing trend is likely the result of multifaceted measures such as improved antibiotic use, improved environmental cleaning, hand hygiene promotion and reinstitution of a single-room policy.

## Disclosure of interest

None declared.

